# Cytotoxic Effects of Alternariol, Alternariol Monomethyl-Ether, and Tenuazonic Acid and Their Relevant Combined Mixtures on Human Enterocytes and Hepatocytes

**DOI:** 10.3389/fmicb.2022.849243

**Published:** 2022-04-22

**Authors:** Danica den Hollander, Celestien Holvoet, Kristel Demeyere, Noémie De Zutter, Kris Audenaert, Evelyne Meyer, Siska Croubels

**Affiliations:** ^1^Laboratory of Pharmacology and Toxicology, Department of Pathobiology, Pharmacology and Zoological Medicine, Faculty of Veterinary Medicine, Ghent University, Merelbeke, Belgium; ^2^Laboratory of Biochemistry, Department of Veterinary and Biosciences, Faculty of Veterinary Medicine, Ghent University, Merelbeke, Belgium; ^3^Laboratory of Applied Mycology and Phenomics, Department of Plants and Crops, Faculty of Bioscience Engineering, Ghent University, Ghent, Belgium

**Keywords:** cytotoxicity, alternariol, alternariol monomethyl-ether, tenuazonic acid, binary and ternary combinations, HepG2 cells, Caco-2 cells, flow cytometry

## Abstract

Alternariol (AOH), alternariol monomethyl-ether (AME), and tenuazonic acid (TeA) are major mycotoxins produced by fungi of the genus *Alternaria* and are common contaminants of food products such as fruits, vegetables, cereals and grains. *Alternaria* mycotoxins are known to cause relevant economic losses and to have a negative impact on human and animal health. EFSA stated in its scientific opinion that data on the toxicity of *Alternaria* mycotoxins in humans and livestock are generally lacking, precluding proper hazard characterization. This study aimed to fill some knowledge gaps by studying the *in vitro* cytotoxicity toward human intestinal epithelial cells (Caco-2) and hepatocytes (HepG2). Cytotoxic properties were assessed by flow cytometric analyses of remaining viable cells (i.e., propidium iodide negative) after mycotoxin exposure for 24–48 h versus solvent control. Treatment of cells with single doses of AOH, AME, and TeA resulted in a dose-dependent loss of cell viability for both cell lines. Half maximal effective concentrations (EC_50_) of the different mycotoxins were comparable for the two cell lines. On HepG2 cells, EC_50_ values varying between 8 and 16, 4 and 5, and 40 and 95 μg/mL were calculated for AOH, AME, and TeA, respectively. On Caco-2 cells, EC_50_ values of 19 μg/mL and varying between 6 and 23, and 60 and 90 μg/mL were calculated for AOH, AME, and TeA, respectively. A general relative cytotoxicity ranking of about 1 = 1 >>> 3 was obtained for AOH, AME, and TeA, respectively. Treatment of both cell lines with combined binary and ternary mixtures of AOH, AME, and TeA in a 1:1:3 ratio, also showed a dose-dependent decrease in cell viability. For both cell lines, the binary combination of especially AME and TeA (1:3 ratio) but also of AOH and AME (1:1 ratio) significantly increased the cytotoxicity compared to the single compound toxicity, although mainly at the highest concentrations tested. The ternary combinations of AOH, AME, and TeA induced only a slight increase in cytotoxicity compared to the single mycotoxins, again at the highest concentrations tested.

## Introduction

Mycotoxins are low-molecular weight secondary metabolites of different fungal genera, able to cause various diseases in human and animals, thus contributing to economic losses in agriculture and stockbreeding. Mycotoxins can be found in various food and feed commodities, especially in grains and grain-based products, but also in vegetables, fruits and fruit juices, oil seeds and oils, spices, coffee, and wine (Bennett and Klich, 2003). The most prevalent mycotoxins, being aflatoxins (e.g., aflatoxin B1, AFB1), fumonisins (FBs), zearalenone (ZEN), type B trichothecenes (e.g., deoxynivalenol, DON), type A trichothecenes (e.g., T2-toxin, diacetoxyscirpenol), and ochratoxin A (OTA) (Gruber-Dorninger et al., 2019), have already been researched widely *in vitro* and *in vivo*, thus either maximum or guidance values for food and feed have been published by EFSA. Alternariol (AOH), alternariol monomethyl-ether (AME), and tenuazonic acid (TeA) are produced in food and feed by fungi of the genera *Alternaria* spp. These *Alternaria* mycotoxins belong to the group of so-called “emerging” mycotoxins, which are frequently detected in food and feed materials, but for which a thorough risk assessment is mostly lacking. Hence, no legal maximum or guidance levels have been established yet for these emerging mycotoxins. Accordingly, EFSA stated that the knowledge of the toxicological properties of *Alternaria* mycotoxins is insufficient for a proper health risk assessment (EFSA, 2011). Furthermore, EFSA has assessed the dietary exposure in the European population to *Alternaria* mycotoxins. The highest exposure of AOH was estimated in toddlers [means of 3.8–71.6 ng/kg bodyweight (BW) per day]. Fruit and fruit products were the most common contributors to the dietary exposure of AOH. Toddlers were also estimated to be exposed the most to AME (means of 3.4–38.8 ng/kg BW per day), and vegetable oil and apples were the main contributors to the dietary exposure. Concerning TeA, the exposure of toddlers was calculated at means of 100–1,614 ng/kg BW per day through cereal-based food for infants and young children (EFSA, 2016). Consequently, the threshold of toxicological concern (TTC) is currently set at 2.5 ng/kg BW per day for AOH and AME, while for TeA it is as high as 1,500 ng/kg BW per day.

Most studies published up to today focus on the toxicity of single *Alternaria* mycotoxins. However, the simultaneous contamination with AOH, AME, and TeA is ubiquitous in several food and feed commodities. Especially grain samples are most frequently contaminated with TeA (15–100%), followed by AOH (2.4–31%) and AME (3–26%) (Fraeyman et al., 2017). When co-occurring, AOH, AME, and TeA are present in a wide range of concentrations. The co-occurrence of *Alternaria* mycotoxins has recently been reviewed (Crudo et al., 2019). Most of the investigated food commodities are found to be simultaneously contaminated with both AOH and AME in approximately the same concentrations ranges (1:1 ratio). TeA on the other hand typically occurs in higher concentrations when compared to AOH and AME (Fraeyman et al., 2017; Crudo et al., 2019), either alone or in the presence of AOH and AME. Along with simultaneous occurrence of *Alternaria* mycotoxins, additive or even synergistic effects of these mycotoxins are possible. Additive effects of the toxicity of AOH and AME (1:1 ratio) have already been observed *in vitro* (Bensassi et al., 2015). AOH has also been shown to be more toxic in the presence of 3- and 15-acetyl-DON (Juan-Garcia et al., 2016). Moreover, mycotoxins with similar modes of action, as it is the case for AOH and AME, are expected to have additive effects (Riley, 1998; Speijers and Speijers, 2004). Nevertheless, to date very little is known about either the *in vitro* nor the *in vivo* toxicity of mixtures of *Alternaria* mycotoxins. As stated by EFSA, there is need for further research to thoroughly assess the risk of these *Alternaria* mycotoxins and their combined mixtures for public health (EFSA, 2011). Therefore, the current study aimed to partly fill these knowledge gaps by further elucidating the cytotoxicity of AOH, AME, and TeA and their binary and ternary combinations (1:1:3 ratio) *in vitro* on relevant human cell lines.

## Materials and Methods

### Chemicals

**FIGURE 1:**
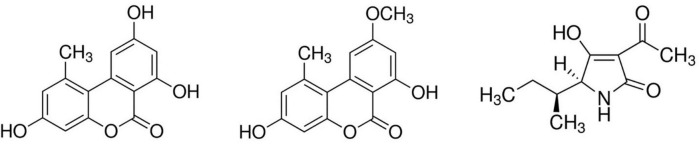
Chemical structures of alternariol (left), alternariol monomethyl-ether (middle), and tenuazonic acid (right) (adapted from: Sigma-Aldrich, MO, United States).

Alternariol, AME, and TeA were purchased from Fermentek (Jerusalem, Israel) and their chemical structures are depicted in Figure 1. Stock solutions of 5 mg/mL were prepared in dimethylsulfoxide (DMSO). All stock solutions were stored at −20°C. Propidium iodide (PI, stored at 4°C protected from light), trypsin-EDTA, antimycotic (amphotericin B) and antibiotic (penicillin and streptomycin) solutions (stored at −20°C) were purchased from Sigma-Aldrich (Overijse, Belgium). All other reagents [Dulbecco’s Modified Eagle Medium (DMEM), Hank’s buffered salt solution (HBSS), and fetal bovine serum (FBS)] for cell culture media were purchased from Thermo Fisher Scientific (MA, United States). TruCount™ beads were purchased from BD Bioscience for flow cytometric cell count and dissolved in phosphate-buffered saline or PBS (Thermo Fisher) containing 1% of Bovine Serum Albumin (BSA, Sigma-Aldrich).

### Cell Cultures

Caco-2 cells and HepG2 cells were purchased from ATCC (VA, United States). The culture medium of Caco-2 consisted of DMEM (high glucose) with 4 mM L-glutamine, 1% non-essential amino acids (NEAA), 10% FBS and 1% of an antibiotic and antimycotic solution (10,000 units penicillin, 10 mg streptomycin, and 25 μg amphotericin B per mL). For HepG2 cells, the composition of the culture medium was DMEM (low glucose) with 10% FBS and 1% of the antibiotic and antimycotic solution, as described above. All cells were cultured in a humified incubator (37°C, 5% CO_2_). To check bacterial and specific mycoplasma contamination, culture media of above cells were tested biweekly using HEK Blue cells in combination with the PlasmoTest™ kit (Invivogen).

### Cytotoxicity Assays and Flow Cytometric Measurements

For both cell lines, HepG2 and Caco-2, 10^5^ cells were resuspended in 1 mL of culture medium, transferred onto a 24-well plate (non-coated, polystyrene, γ-sterilized, SPL Life Sciences, South Korea) and incubated for 72 h to obtain proliferating cells. After 3 days, cells were washed with HBSS in order to remove dead cells and cell debris. The final DMSO concentrations of 1.2–2.4% were found to be non-cytotoxic in preliminary experiments. Stock solutions of AOH, AME, and TeA (5 mg/mL in DMSO) were diluted with culture medium to standard solutions of 120, 60, 20, 10, 5, 1, 0.5, 0.25, and 0.1 μg/mL. For single mycotoxin experiments, each of the different concentrations was added to the 24-well plates in triplicate or quadruplicate (technical replicates). For mycotoxin combination experiments, AOH, AME, and TeA were added in binary and ternary combinations in a 1:1:3 ratio, respectively, at the following concentration ranges: 1/1/3, 2/2/6, 3/3/9, and 4/4/12 μg/mL of AOH/AME/TeA; 1/1, 2/2, and 4/4 μg/mL of AOH/AME; 1/3, 2/6, and 3/9 μg/mL of AOH/TeA and 1/3, 2/6, and 3/9 μg/mL of AME/TeA. Finally, DMSO was added to obtain an equal DMSO concentration (1.2 or 2.4%) for each tested mycotoxin concentration. Also, 3 or 4 wells containing only cells in culture medium as a negative control, H_2_O_2_ (1 or 2 mM) as positive control, and 1.2 and 2.4% DMSO as a solvent control were included. The 24-well plates were incubated for 24 (HepG2) and 48 h (Caco-2) at 37°C and 5% CO_2_. After incubation, the medium was removed and the remaining cells on the bottom were trypsinized (0.25% trypsin and 0.02% sodium EDTA in Hanks’ Balanced solution with phenol red, during 10 min). Each well was diluted with culture medium, and the resuspended cells were transferred into Eppendorf cups and centrifuged for 5 min at 300 × *g*. The supernatant was removed and the cell pellets were resuspended in 200 μL of HBSS and transferred to a 96-well plate, which was again centrifuged for 5 min at 1,800 × *g*. The latter washing and centrifugation steps were repeated once. Subsequently, 180 μL of the PI solution (1 μg/ml in PBS) was added to the wells for staining. PI stains the DNA of dead cells with a damaged plasma membrane, identifying merely the late apoptotic/necrotic cells. To check autofluorescence of the cells, wells without PI were included. Last, 20 μL of a TruCount™ beads mixture was added to the wells and mixed for 5 min on a plate shaker (100 rpm). Measurement of the cytotoxicity was performed with a Cytoflex flow cytometer (Beckman Coulter Life Sciences, IN, United States) within 30 min after cell staining. The sample flow-rate was set at 60 μL/min; stopping rules were limited to either 150 s or 200 beads. Cell fluorescence was excited at 488 nm, and PI fluorescent emission was measured using an emission filter of 585 ± 21 nm. All acquired data were processed using CytExpert software (v2.0.0.153, Beckman Coulter, Inc., CA, United States). For all samples, the single cell population was gated on Forward Scatter light (FSC)-Area versus FSC-Height scatter plot to exclude aggregates and the debris fraction. Viable and dead single cells were identified as PI− and PI+, respectively. Using TruCount™ beads as an internal control, the absolute amount of counted events/μL cells, reassuring a correct count of viable cells, was calculated by using following formula:


$$A=\frac{X}{Y}\times \frac{N}{V}$$

with *A*, the absolute count of the cell population; *X*, the number of cell events; *Y*, the number of bead events; *V*, test volume; and *N*, number of beads per well.

### Data Processing and Statistical Analysis

Flow cytometric results were shown as histograms by the CytExpert software with two peaks, i.e., the viable cells (PI−) versus dead cells (PI+). The normalized percentage of viable cells was calculated using the number of viable single cells (percentage of PI− cells multiplied by the counted events/μL) per well remaining after incubation with mycotoxins versus the number of viable cells (percentage of PI− cells multiplied by the counted events/μL) per well remaining after incubation with DMSO as solvent control. To reassure the data was normally distributed the Skewness and Kurtosis *z*-values were calculated in SPSS (IMB, NY, United States). Statistical differences between groups (different concentrations of single and combined mycotoxins) were compared using one-way ANOVA analysis and the Tukey test for pairwise multiple comparisons. A *p*-value less than 0.05 was considered statistically significant.

To obtain reliable EC_50_ values, each experiment, per mycotoxin and per cell line, was performed in triplicate (biological replicates) with each 3–4 technical replicates, depending on the cell yield per experiment. For each biological replicate a dose-response curve was created with the concentrations of mycotoxins in log scale on the *x*-axis and the percentage of viable cells after mycotoxin incubation of each technical replicate on the *y*-axis. All dose-response curves were expanded toward the values of the positive control per biological replicate. A gradient or slope non-linear curve fitting was applied, and using following formula:


$${\text{EC}}_{50}=\frac{\min+\left(\max-\min\right)}{1+10^{n\,\left(\log10\,\left(x\right)-\log10\,\left(\text{EC}\,50\right)\right)}}$$

An EC_50_ value was calculated, using the Solver Add-in function in Microsoft Excel (Redmond, WA, United States), for each biological replicate, when the given data allowed the calculations.

When possible, for each dose-response curve, plotted in linear ln-scale, prediction and confidence intervals (95%) around the best fitted regression model were calculated using the R-packages *stats* and *investr* (function *nls* resp. *PredFit*) (Greenwell and Kabban, 2014; R-Core-Team., 2020). The lowest concentration inducing a significant decrease in cell viability (LOEL or Lowest Observed Effect Level) was set at the intersection of the lower value of the 95% prediction interval. The NOEL (No Observed Effect Level) was the tested concentration that fell into the 95% prediction interval.

## Results

### Single Mycotoxin Experiments on HepG2 Cells

**FIGURE 2:**
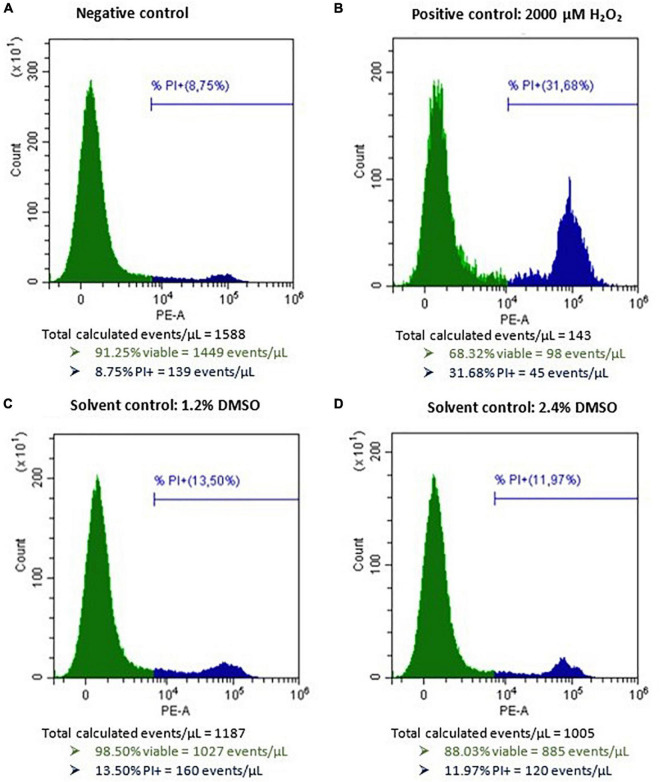
Histograms of HepG2 cells after flow cytometric analysis cells after exposure to **(A)** only cell culture medium as negative control, **(B)** 2,000 μM H_2_O_2_ as a positive control, **(C)** 1.2% DMSO as a solvent control, and **(D)** 2.4% DMSO as second solvent control for 48 h. PI, propidium iodide. The green peak indicates the viable cells (PI–), the blue peak represents the dead cells (PI+).

**FIGURE 3:**
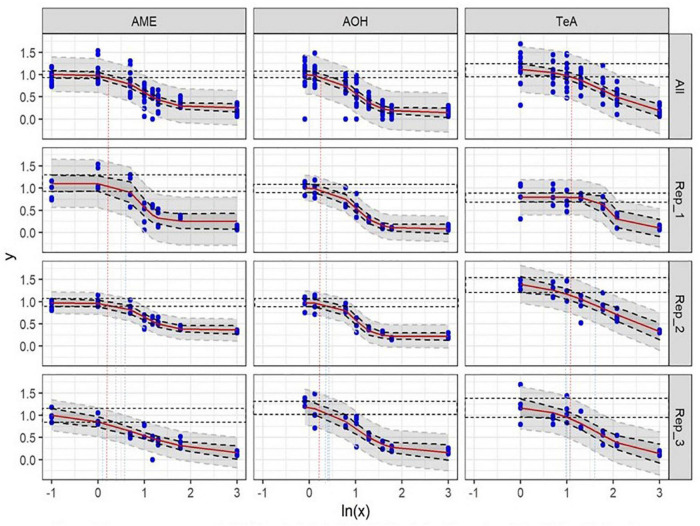
Dose-response curves of all (All) and of the individual biological replicates (Rep_1, Rep_2, and Rep_3) from the cytotoxicity assays of alternariol (AOH), alternariol monomethyl-ether (AME), and tenuazonic acid (TeA) on HepG2 cells. The *x*-axis shows the ln-values of the different mycotoxin concentrations and the *y*-axis the percentage of viable cells (PI–) after mycotoxin exposure in relation to the viable cells of the solvent control (DMSO). A gradient or slope non-linear curve fitting was applied. The prediction and confidence intervals are indicated by the dark and light gray zone, respectively. Lowest-observed-effect level (LOEL) values are indicated as blue (single replicates) and red (all replicates) dotted lines, respectively. All biological replicates consist of at least three technical replicates.

After incubation of HepG2 cells for 24 h with 2 mM H_2_O_2_ as a positive control, an average percentage of 18.5 ± 9.0% (mean ± SD, *n* = 24) of viable cells (PI−) was measured (normalized data), confirming H_2_O_2_ to be a reliable inducer of cell death on HepG2 cells. Incubation of HepG2 cells with either 1.2 or 2.4% DMSO for 48 h as a solvent control resulted in 1,838 ± 646 and 1,693 ± 655 viable cells per μL (mean ± SD), respectively (data not normalized). In comparison, the viable cell count per μL measured for the negative and positive control conditions (*n* = 24) were 1,831 ± 504 and 352 ± 241, respectively (data not normalized). Representative flow cytometric results of the negative, positive, and solvent control conditions of HepG2 cells prior to normalization are presented in Figure 2. No autofluorescence was observed. The dose-response curves of the three individual biological replicates of AOH, AME, and TeA on HepG2 normalized toward DMSO are presented in Figure 3 (Rep_1, Rep_2, and Rep_3). To diminish the confidence interval and thus improve the accuracy of the EC_50_ values, all biological replicates per mycotoxin were merged (All). Increasing concentrations of AOH, AME, and TeA resulted in a decline of the normalized percentage of viable cells.

In all three biological replicates, an AOH concentration of 1.0 **±** 0.0 μg/mL (mean ± SD) did not induce any cytotoxic effect on HepG2 cells after 24 h of incubation (NOEL). The LOEL after 24 h of incubation was found to be 1.436 ± 0.038 μg/mL (mean ± SD). Slightly different EC_50_ values were obtained for the three biological replicates due to biological inter-experimental variability, i.e., 11.14, 7.93, and 15.98 μg/mL, respectively, and resulting in an overall EC_50_ value of 11.68 **±** 4.05 μg/mL (mean ± SD).

Also for AME a concentration of 1.0 ± 0.0 μg/mL (mean ± SD) did not induce any cytotoxic effect on HepG2 Cells after 24 h of incubation (NOEL). Additionally, the LOEL was 1.492 ± 0.330 μg/mL (mean ± SD). Comparable EC_50_ values were obtained for the three biological replicates of 5.33, 5.40, and 4.47 μg/mL, respectively, resulting in an overall EC_50_ value of 5.07 ± 0.52 μg/mL (mean ± SD).

In contrast, TeA did not induce statistically relevant decreases in cell viability after 24 h incubation until concentrations reached 3.072 ± 0.613 μg/mL (mean ± SD) (NOEL). The LOEL was calculated at 3.690 ± 0.113 μg/mL (mean ± SD). A more pronounced variability resulted in EC_50_ values of 89.38, 95.34, and 40.60 μg/mL, respectively, for the three biological replicates or an overall EC_50_ value of 75.11 ± 30.03 μg/mL (mean ± SD).

### Single Mycotoxin Experiments on Caco-2 Cells

**FIGURE 4:**
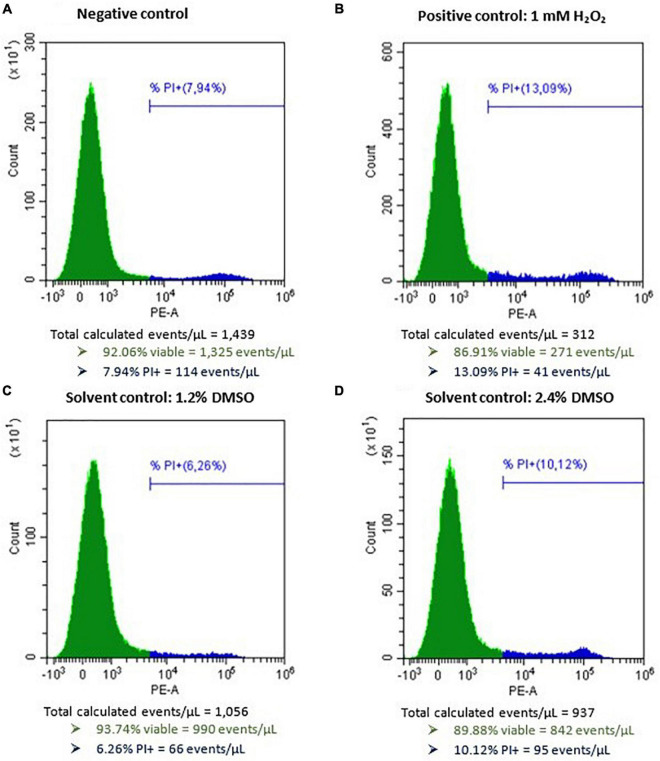
Histograms of Caco-2 cells after flow cytometric analysis following exposure to **(A)** only cell culture medium as negative control, **(B)** 1 mM H_2_O_2_ as a positive control, **(C)** 1.2% DMSO as a solvent control, and **(D)** 2.4% DMSO as second solvent control, for 48 h. PI, propidum iodide. The green peak indicates the viable cells (PI–), the blue peak represents the dead cells (PI+).

**FIGURE 5:**
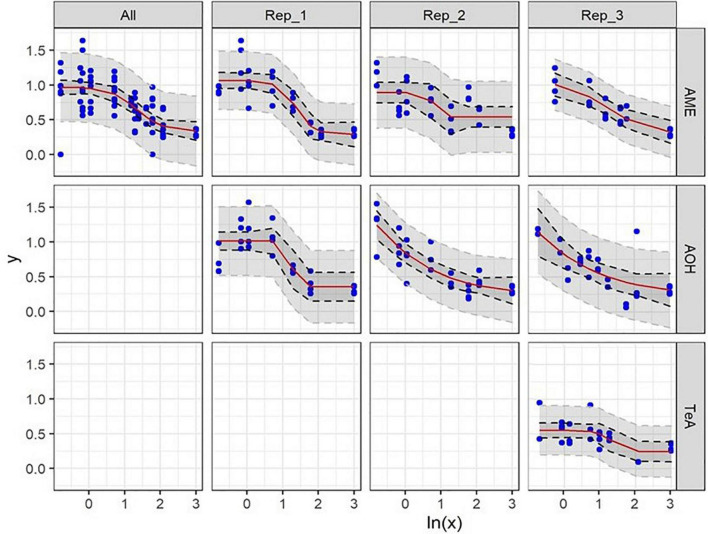
Dose-response curves of all (All) and of the individual biological replicates (Rep1_, Rep2_, and Rep3) from the cytotoxicity assays of alternariol (AOH), alternariol monomethyl-ether (AME), and tenuazonic acid (TeA) on Caco-2 cells. The *x*-axis shows the ln-values of the different mycotoxin concentrations and the *y*-axis the percentage of viable cells (PI–) after mycotoxin exposure in relation to the viable cells of the solvent control (DMSO). A gradient or slope non-linear curve fitting was applied. The prediction and confidence interval are indicated by the dark and light gray zones, respectively. No reliable LOEL values could be calculated. All biological replicates consist of at least three technical replicates.

After incubation of Caco-2 Cells with 1 mM H_2_O_2_ as a positive control for 48 h, a mean (±SD) percentage of 43.7 ± 15.2 (*n* = 14) viable cells (PI−) was observed (normalized data). Incubating Caco-2 cells with DMSO as a solvent control for 48 h (1.2 and 2.4%) resulted in 736 ± 256 and 615 ± 183 viable cells per μL (*n* = 14), respectively (data not normalized). The average (±SD) viable cell count per μL measured for the negative control for Caco-2 cell experiments was 899 ± 265 and for the positive control conditions 281 ± 60 (data not normalized). No autofluorescence was observed. Representative flow cytometric results of the negative, positive, and solvent control conditions of Caco-2 cells prior to normalization are presented in Figure 4. The dose-response curves of the three biological replicates of AOH, AME, and TeA on Caco-2 cells are presented in Figure 5. Since a large inter- and intra-experimental variability was observed during Caco-2 experiments, no prediction intervals could be established for some biological replicates; hence, these dose-response curves are missing. On Caco-2 cells, increasing concentrations of AOH, AME, and TeA resulted in a dose-dependent decrease in the percentage of viable cells as well.

Due to a more pronounced inter- and intra-experimental biological variability, not each biological replicate resulted in a reliable EC_50_ value for the three mycotoxins on Caco-2 cells. Also, no average mycotoxin concentrations that did not induce any cytotoxic effect nor LOEL could be calculated for any of the three mycotoxins. For the first biological replicate of AOH, containing four technical replicates, an EC_50_ value of 18.71 μg/mL was obtained on Caco-2 cells. No EC_50_ values could be calculated for the second and third biological replicate due to a non-consistent dose-dependent decrease in cell viability. The cytotoxicity of AME cells was comparable to that of AOH, as for HepG2 cells. The obtained EC_50_-values, being 23.12, 6.1, and 16.94 μg/mL for the three biological replicates, resulted in an overall EC_50_ value of 15.38 ± 8.62 μg/mL (mean ± SD). The EC_50_ values of TeA on Caco-2 cells revealed a more pronounced inter-experimental variability with values of 59.90, 60.51, and 90.21 μg/mL, or an overall EC_50_ value of 70.22 ± 17.32 μg/mL (mean ± SD) and was thus comparable to that on HepG2 cells.

### Combined Mycotoxin Experiments on HepG2 and Caco-2 Cells

Since the single mycotoxin experiments revealed an approximate toxicity ranking of 1 = 1 >>> 3 for AOH, AME, and TeA, a ratio of 1:1:3 for the combined mycotoxin experiments was applied. Mycotoxin combinations and concentrations that induced a significant increase in cytotoxicity on HepG2 and Caco-2 cells in comparison with their single doses are presented in Table 1.

**TABLE 1:** Overview of the mycotoxin combinations and concentrations of alternariol (AOH), alternariol-monomethyl ether (AME), and tenuazonic acid (TeA) that induced statistically significant decreases in cell viability when compared to the single doses after ANOVA analysis and Tukey *post hoc* testing.

Compared mycotoxin combinations				*p*-Value	
Mycotoxin	Concentration in μ g/mL	Mycotoxins	Concentration in μ g/mL	HepG2-cells	Caco-2 cells
AME	4	AOH – AME	4–4	<0.01	<0.01
AME	4	AOH – AME – TeA	4–4–12	0.03	<0.01
TeA	12	AOH – AME – TeA	4–4–12	/	<0.01
TeA	12	AOH – TeA	4–12	/	0.02
AME	2	AME – TeA	2–6	<0.01	/
	4		4–12	<0.01	<0.01
TeA	3	AME – TeA	1–3	0.01	/
	6		2–6	<0.01	/
	12		4–12	/	<0.01

**FIGURE 6:**
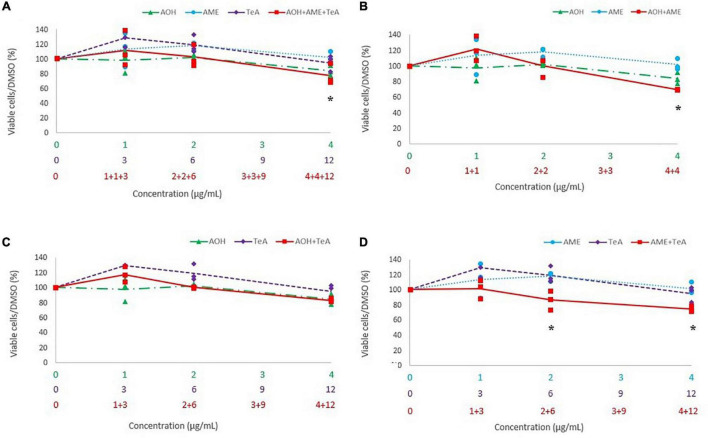
Comparison of the percentage of viable cells after 24 h of exposure to a **(A)** ternary mixture of alternariol (AOH) (green triangle), alternariol monomethyl-ether (AME) (blue circle), and tenuazonic acid (TeA) (purple diamond), **(B)** binary mixture of AOH and AME, **(C)** binary mixture of AOH and TeA, **(D)** binary mixture of AME and TeA, to the single mycotoxin exposure of AOH, AME, and TeA on HepG2 cells. The primary *x*-axis shows the single concentration of AOH, AME, or TeA in μg/mL, while the secondary and ternary *x*-axis show the combined mycotoxin concentrations in μg/mL. Total mycotoxin concentrations differs per *x*-axis. The *y*-axis shows the percentage of viable cells after mycotoxin exposure. Results are presented as an average (full, dotted, or half dotted line) from three biological replicates, each representing four technical replicates. Asterisks indicate significant differences after ANOVA analysis and Tukey *post hoc* test.

Figure 6A shows the cytotoxicity of the combinations of AOH, AME, and TeA (1:1:3 ratio) versus their effect as single mycotoxins on HepG2 cells. Figures 6B–D show the cytotoxic effects of the binary combinations of either AOH and AME (1:1 ratio), AOH and TeA (1:3 ratio), or AME and TeA (1:3 ratio), in comparison to their single mycotoxin exposure on HepG2 cells. The combined mixture of AOH (4 μg/mL) and AME (4 μg/mL) induced significantly more cell death than the single dose of AME (4 μg/mL) (*p* < 0.01). After exposure of HepG2 cells to the combined ternary mixture of AOH (4 μg/mL), AME (4 μg/mL), and TeA (12 μg/mL) a significant lower cell viability was observed compared to the single dose of AME (4 μg/mL) (*p* = 0.03). The combined mixture of AME and TeA showed strongest effects, since the binary mixture (1 and 3 μg/mL; 2 and 6 μg/mL; 4 and 12 μg/mL) decreased the cell viability significantly compared to both, the single doses of AME (2 and 4 μg/mL) and TeA (3 and 6 μg/mL). No differences were observed for the remaining mycotoxin combinations and concentrations on HepG2 cells.

**FIGURE 7:**
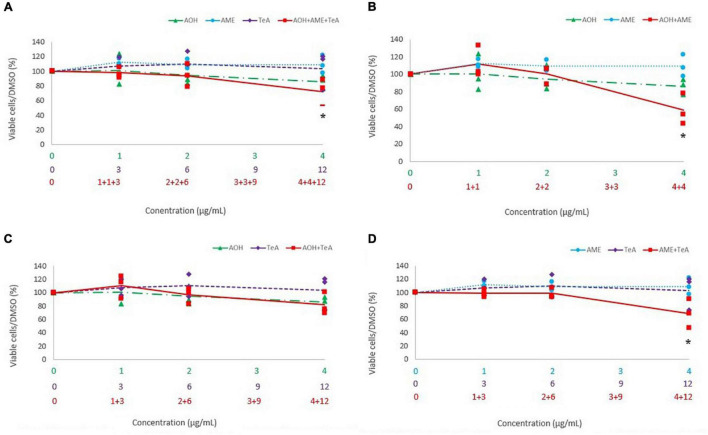
Comparison of percentage of viable cells after 48 h of exposure to a **(A)** ternary mixture of alternariol (AOH) (green triangle), alternariol monomethyl-ether (AME) (blue circle), and tenuazonic acid (TeA) (purple diamond), **(B)** binary mixture of AOH and AME, **(C)** binary mixture of AOH and TeA, **(D)** binary mixture of AME and TeA, to the single mycotoxin exposure of AOH, AME, and TeA on Caco-2 cells. The primary *x*-axis shows the single concentration of AOH, AME, or TeA in μg/mL, while the secondary and ternary *x*-axis show the combined mycotoxin concentrations in μg/mL. Total mycotoxin concentrations differ per *x*-axis. The *y*-axis shows the percentage of viable cells after mycotoxin exposure. Results are presented as an average (full, dotted, or half dotted line) from three biological replicates, each representing four technical replicates. Asterisks indicate significant differences after ANOVA analysis and Tukey *post hoc* host.

Figure 7A shows the cytotoxic effects of the ternary combination of AOH, AME, and TeA (1:1:3 ratio) versus their effect as single mycotoxins on Caco-2 cells. Figures 7B–D show the cytotoxic effects of the binary combinations of either AOH and AME (1:1 ratio), or AOH and TeA (1:3 ratio), or AME and TeA (1:3 ratio), respectively, in comparison to their single mycotoxin exposure on Caco-2 cells. Firstly, the binary mixture of AOH and AME (4 and 4 μg/mL) induced significantly more cell death compared to the single dose of AME (4 μg/mL; *p* < 0.01). Also, after exposure to the ternary mixture of AOH, AME, and TeA (4, 4, and 12 μg/mL, respectively), a significant higher decrease in cell viability was observed in comparison to the single doses of AME (4 μg/mL; *p* < 0.01) and TeA (12 μg/mL; *p* < 0.01). Also the binary mixture of AOH and TeA (4 and 12 μg/mL) revealed higher cytotoxic effects compared to the single dose of TeA. In accordance to HepG2 cells, the binary mixture of AME and TeA induced strongest cytotoxic effects, since the combination of 4 and 12 μg/mL of AME and TeA induced an increasing cytotoxicity in comparison to the both the single doses of AME and TeA, respectively. For the remaining combined mixtures, no differences were observed on Caco-2 cells.

## Discussion

To the authors’ knowledge, effects of single mycotoxins AOH, AME, and TeA as well as their binary and ternary combinations were at first examined by flow cytometry. Although the *in vivo* concentrations of AOH, AME, and TeA at the level of human enterocytes and hepatocytes are not yet established, the contamination levels in food and feed are well documented (Fraeyman et al., 2017; Crudo et al., 2019). More specifically, 82, 80, and 62% of feed and feed ingredient samples collected in Europe were contaminated with AOH, AME, and TeA, respectively. However, prevalence and concentration levels of food and feed ingredients vary substantially, since AOH, AME, and TeA occurred in unprocessed cereals in 2.4–47, 3.1–7.1, and 15–68% of the samples, and in concentrations ranging from 0.75 to 832, 0.3 to 905, and 0.1 to 4,224 μg/kg, respectively; while for tomato products, 28–71, 20–79, and 40–100% of the samples were contaminated with AOH, AME, and TeA at concentrations ranging between 2 and 41.6, 0.9 and 7.8, and 5 and 462 μg/kg, respectively. Once ingested, these mycotoxins are able to pass through the intestinal barrier and reach the liver, leaving enterocytes and hepatocytes as their main potential target cells. Therefore, this study aimed to further elucidate the cytotoxicity of *Alternaria* mycotoxins on human enterocytes and hepatocytes by determining valuable toxicological endpoints (EC_50_ values) on these relevant human cell lines.

It has been reported that AOH and AME are cytotoxic, inducing apoptotic cell death through the mitochondrial pathway (Bensassi et al., 2011, 2012; Fernandez-Blanco et al., 2016b). The current research confirmed AOH and AME to be cytotoxic in a dose-dependent manner on HepG2 and Caco-2 cell lines. Studying the mechanism of cell death was not part of this PI-based cytotoxicity flow cytometric assay. In addition, AOH was found to form reactive oxygen species (ROS) and to interact with DNA topoisomerase, inducing single and double strand breaks. A decrease in cell proliferation was observed after exposure to AOH, due to cell cycle arrest in the G2/M-phase. Similarly, AME was found to be mutagenic by causing DNA strand breaks and cell cycle arrest (Fehr et al., 2009; Bensassi et al., 2012; Fleck et al., 2012, 2016; Fernandez-Blanco et al., 2016b; Solhaug et al., 2016a,b). In metabolically active human hepatocytes (HepaRG), AOH, and AME did not induce any cytotoxicity at concentrations up to 100 μM (27.2 and 25.8 μg/mL, respectively) as assessed by the MTT [3-(4,5-dimethylthiazol-2-yl)-2,5-diphenyltetrazolium bromide] assay (Hessel-Pras et al., 2019). However, on lower metabolically active HepG2 cells, AME induced stronger cytotoxic effects compared to AOH with significant decreases in cell viability of 68 ± 7, 35 ± 2, and 30 ± 8% after 24 h exposure to 10, 50, and 100 μM (2.58, 12.91, and 25.8 μg/mL), respectively (Hessel-Pras et al., 2019). The current study corroborated these findings, since AME was also found to induce highest cytotoxicity on HepG2 cells with an EC_50_ value of 5.07 ± 0.52 μg/mL compared to 11.68 ± 4.05 μg/mL for AOH. Even though the lowest concentrations inducing cytotoxicity on HepG2 cells were comparable for AOH and AME (1.436 ± 0.038 and 1.492 ± 0.330 μg/mL, respectively), a more rapid decrease in cell viability was observed after 24 h of incubation with AME, when compared to AOH. The obtained EC_50_ value for AME (5.07 ± 0.52 μg/mL) again corroborates the study of Hessel-Pras et al. (2019), observing the concentration which induced a 50% loss in cell viability to lie between 2.58 and 12.91 μg/mL. On Caco-2 cells the EC_50_ value of AME of 15.38 ± 8.62 μg/mL obtained in the current study was higher than for HepG2 cells. However, due to the difference in exposure time, being 48 h for HepG2 and 24 h for Caco-2, comparisons in sensitivity need to be interpreted carefully. An EC_50_ value of 31 μg/mL was reported by Bensassi et al. (2011) after incubation of AME for 24 h on human colon carcinoma cells (HCT116 cells), as assessed by flow cytometric analysis. In the study of Hessel-Pras et al. (2019), AOH caused a significant decrease in cell viability to 53 ± 13% in HepG2 and 66 ± 17% in HepaRG cells after 24 h exposure to 27.3 μg/mL. This study set an EC_50_ value range of 7.93–15.98 μg/mL for AOH on HepG2 cells and of 18.71 μg/mL on Caco-2 cells. Accordingly, Bensassi et al. (2012) found an EC_50_ value for AOH of 18 μg/mL after exposure of HT-29 cells for 24 h, assessed by flow cytometry. On the other hand, Fernandez-Blanco et al. (2014) were not able to observe a 50% loss of cell viability on Caco-2 cells with either MTT, or neutral red (NR) or phosphatidylcholine colorimetric (PC) assay after exposure of AOH at concentrations up to 27 μg/mL. NOEL and LOEL values of approximately 1 and 1.5 μg/mL were found for AOH and AME on HepG2-cells in this study, respectively. To the authors’ knowledge, no NOEL or LOEL values for AOH, AME, or TeA have been reported in literature as such. Nevertheless, the LOEL levels found in this study can be compared to the concentrations found in other studies of *Alternaria* mycotoxins, e.g., Bensassi et al. (2011, 2012), who found AOH and AME to be cytotoxic on Caco-2 cells, starting from concentrations of 10 μM (2.7 and 2.6 μg/mL, respectively). However, it has to be mentioned that these concentrations are dependent from the chosen concentration ranges, as was the NOEL value in the current study.

Differing from AOH and AME, TeA exerts its toxicity by inhibition of the protein release from ribosomes (Shigeura and Gordon, 1963). In the study of Hessel-Pras et al. (2019), TeA induced a significant decrease in cell viability to 56 ± 13% after 24 h exposure to 19.72 μg/mL on HepG2 cells. In this study, the lowest EC_50_ value of TeA was found to be 40.60 μg/mL, while the highest EC_50_ value was as high as 95.34 μg/mL, indicating TeA to be about five times less toxic than AOH and AME on HepG2 cells. Taking the difference in incubation time into account, a similar overall EC_50_ value of 70.22 ± 17.32 μg/mL (mean ± SD) was found on Caco-2 cells, indicating TeA to be about three times less toxic than AOH and AME. Both EC_50_ values are in accordance with literature, claiming TeA to be less cytotoxic compared to the other *Alternaria* mycotoxins (Pero et al., 1973; Zhou and Qiang, 2008; Schwarz et al., 2012). Zhou and Qiang (2008) found EC_50_ values ranging from 41.64 to 85.98 μg/mL for TeA after incubation of 24 h, in line with the results obtained in the current research. Both the cell line and the cytotoxicity assay in the latter differed from the present research, since Zhou and Qiang (2008) assessed 3T3, CHL, and L-O2 cells by an MTT assay. Moreover, Vejdovszky et al. (2016) could only detect cytotoxic effects of TeA on Caco-2 cells, being a decreased mitochondrial activity to 68%, at comparably high concentrations of 50 μg/mL, again corroborating the observations in the current study. The NOEL and LOEL levels for TeA found in this study were approximately 3 and 3.5 μg/mL, respectively. Analogously as for AOH and AME, no NOEL and LOEL values have been reported for TeA as such, yet. However, LOEL value found in this study can be compared to the lowest concentration of 12.5 μg/mL, tested by Zhou and Qiang (2008), which already inhibited cell proliferation on 3T3, CHL, and L-O2 cells.

Regarding the toxicokinetic properties of *Alternaria* mycotoxins, for AOH, a low systemic absorption was reported in mice with 90% of the total dose (200 and 1,000 mg/kg BW were evaluated) being excreted in the feces and 9% in urine, while plasma levels did not exceed 0.06% at 24 h post administration, identifying the gastro-intestinal tract as main target of AOH (Schuchardt et al., 2014). Moreover, four metabolites, namely 2-, 4-, 8-, and 10-hydroxy-AOH were detected in blood and urine, implying phase I metabolization to be partly responsible for the low oral bioavailability. A recently published study investigated the metabolism and excretion of 17 *Alternaria* toxins in rats (Puntscher et al., 2019). Out of the administered doses of AOH, AME, and TeA (up to 12, 10, and 8,945 μg/kg BW, respectively), 9, 87, and 0.3% were recovered in urine and feces, respectively, showing clear differences in toxicokinetic properties between the three individual mycotoxins. Also, a phase II metabolite of AME, AME-3-sulfate, has been determined in urine and represented 23% of the AME intake. In contrast, an effective passage of AOH and its metabolites across the intestinal monolayer has been observed *in vitro* in Caco-2 cells (Burkhardt et al., 2009). In the same study, only a small percentage of two AME metabolites were able to cross the intestinal epithelium, whereas no unconjugated AME reached the basolateral compartment. Generally, once the mycotoxins cross the gastrointestinal barrier, the liver will be the next site of action. However, according to Hessel-Pras et al. (2019) knowledge of possible adverse effects of *Alternaria* mycotoxins on liver cells is limited. The current research proved the cytotoxic properties of AOH and AME to be evenly toxic or even more toxic on hepatocytes as was demonstrated on enterocytes, with EC_50_ values for AOH and AME of 11.68 ± 4.05 and 5.07 ± 0.52, and of 18.71 and 15.38 ± 8.62 μg/mL on HepG2 and Caco-2 cells, respectively. TeA has already been shown to be almost completely intestinally absorbed by pigs and broiler chickens (Fraeyman et al., 2015), but no data regarding cellular metabolism of TeA have been published so far.

Hitherto, very little is known regarding the *in vivo* or even the *in vitro* toxicity of combined *Alternaria* mycotoxins. The second objective of the current study was therefore to perform a first characterization of possible synergistic, additive or even antagonistic effects of the three *Alternaria* mycotoxins AOH, AME, and TeA. Studies regarding the cytotoxicity of mycotoxins combinations mostly aim to reveal additive effects at low doses already proven to be either non- or not significantly cytotoxic as single doses. In this context, AOH has been shown to be more cytotoxic on HepG2 cells (MTT assay) in combination with 3- and 15-acetyl-DON due to additive or synergistic effects in comparison to the single mycotoxin dose (Juan-Garcia et al., 2016). AOH and AME were found to induce an additive cytotoxic effect on HCT116 cells assessed by flow cytometric analysis and the interaction index *V* (Bensassi et al., 2015). The latter study compared the single doses (25 μM) of both mycotoxins with their combined dose of each 25 μM (i.e., 50 μM total toxin load). A dose-dependent decrease in cell viability is expected for both mycotoxins when increasing the dose to doses twice as high. In the current study, a similar approach as in Bensassi et al. (2015) was used. Similarly to the results of Bensassi et al. (2015), the binary combinations of AOH and AME (4 and 4 μg/mL, respectively) induced an increased cytotoxic effect compared to the corresponding single doses of AME on HepG2 cells and Caco-2 cells. Especially, the binary mixture of AME and TeA resulted in a stronger cytotoxicity, since combined doses of AME and TeA induced a stronger decrease in cell viability compared to the corresponding single doses of both AME and TeA on both cell lines. More specifically, the combined concentration of 2 μg/mL AOH and 6 μg/mL TeA on HepG2 already induced a significant decrease in cell viability compared to the single compound toxicity, while on Caco-2 cells only the highest concentrations of 4 μg/mL AOH and 12 μg/mL TeA amplified the single compound toxicity, raising the question whether hepatic cells are more prone to binary combinations of these mycotoxins than intestinal cells. Unexpectedly, in another study TeA was found to decrease the toxicity of DON in combined toxicity assays (WST-1) on Caco-2 cells after 24 h of exposure (Vejdovszky et al., 2016). A recent study investigated the interaction of effects of enniatin B, DON, and AOH on Caco-2 cells by means of the MTT assay and concluded that ternary combinations produced a smaller decrease in cell viability compared to binary combinations (Fernandez-Blanco et al., 2016a). This unexpected finding was confirmed in the current study, since ternary combinations of AOH, AME, and TeA also did not induce more cytotoxicity compared to their binary exposure, either on HepG2 cells or on Caco-2 cells, except for the combination of the highest concentrations tested (4 + 4 + 12 μg/mL for AOH, AME, and TeA, respectively).

In this study, intestinal (Caco-2) cells were exposed for 48 h, while hepatic (HepG2) cells were exposed for 24 h, since this is a realistic time period needed for mycotoxins to reach the liver after their intestinal absorption and to exert cytotoxic properties. Indeed, in humans and pigs it takes about 24–96 h for solid food to pass through the entire gastro-intestinal tract (Read et al., 1980; Snoeck et al., 2004). Furthermore, proliferating cells were used, since these cells are known to be more prone to any kind of toxicological agent exposure and thus also to mycotoxins (Broekaert et al., 2016; Fraeyman et al., 2018). Since, especially intestinal, but also hepatic cells are being continuously renewed throughout life, proliferating cells are continuously encountering all kind of xenobiotics or toxins. Hence, this study design reproduces a realistic mycotoxin exposure scenario. Nevertheless, it is not straightforward to extrapolate *in vitro* data to *in vivo* settings.

Concluding, there is an urgent need for more *in vitro* toxicity research of single and combined *Alternaria* mycotoxins to further elucidate possible synergistic effects, sometimes increasing their toxicity and thus impeding human and animal health. As a future prospect, it is also particularly important to identify novel metabolites of the *Alternaria* mycotoxins and their associated metabolic pathways. Moreover, research on the *in vivo* toxicity and toxicokinetic profiling of *Alternaria* toxins is mandatory for a proper risk assessment. In the current research, a valuable toxicological endpoint, namely EC_50_ values for AOH, AME and TeA was defined, paving a way for further *in vitro* cytotoxicity research but especially genotoxicity assays by finetuning selected doses for future testing.

## Data Availability Statement

The raw data supporting the conclusions of this article will be made available by the authors, without undue reservation.

## Author Contributions

SC, EM, KA, and KD conceived and designed the experiments. CH performed the experiments. KA, ND, and DH did the data and statistical analysis. DH drafted the manuscript. All authors read and approved the final version of the manuscript.

## Conflict of Interest

The authors declare that the research was conducted in the absence of any commercial or financial relationships that could be construed as a potential conflict of interest.

## Publisher’s Note

All claims expressed in this article are solely those of the authors and do not necessarily represent those of their affiliated organizations, or those of the publisher, the editors and the reviewers. Any product that may be evaluated in this article, or claim that may be made by its manufacturer, is not guaranteed or endorsed by the publisher.
